# Probiotics on Pediatric Functional Gastrointestinal Disorders

**DOI:** 10.3390/nu10121836

**Published:** 2018-11-29

**Authors:** Anna Pärtty, Samuli Rautava, Marko Kalliomäki

**Affiliations:** 1 Department of Pediatrics, University of Turku, 20521 Turku, Finland; samrau@utu.fi (S.R.); markal@utu.fi (M.K.); 2 Department of Pediatrics and Adolescent Medicine, Turku University Hospital, 20521 Turku, Finland

**Keywords:** probiotics, functional gastrointestinal disorders, functional abdominal pain disorders, functional constipation, infantile colic

## Abstract

The potential association between gut microbiota perturbations and childhood functional gastrointestinal disturbances opens interesting therapeutic and preventive possibilities with probiotics. The aim of this review was to evaluate current evidence on the efficacy of probiotics for the management of pediatric functional abdominal pain disorders, functional constipation and infantile colic. Thus far, no single strain, combination of strains or synbiotics can be recommended for the management of irritable bowel syndrome, functional abdominal pain or functional constipation in children. However, *Lactobacillus reuteri* DSM 17938 may be considered for the management of breastfed colic infants, while data on other probiotic strains, probiotic mixtures or synbiotics are limited in infantile colic.

## 1. Introduction

The role of the intestinal microbiota in health and disease has been the focus of intensive research during the past decades. This interest has largely resulted from studies indicating differences in gut microbiota between healthy individuals and patients afflicted with non-communicable disease. Particularly, various chronic gastrointestinal disorders such as functional gastrointestinal disorders (FGID), colic crying, inflammatory bowel disease, and celiac disease have been associated with perturbations in gut microbiota composition. While these associations offer no proof of causality or direction, they serve as starting points for research aiming to establish whether gut microbiota disturbances might predispose one to or be involved in the causal complex leading to disease. The association between dysbiosis and functional gastrointestinal disorders in children and infants has raised great interest in modulating the gut microbiota composition and activity as a promising therapeutic and preventive option. The aim of this review was to evaluate current evidence on the efficacy of probiotic interventions for the management and prevention of functional gastrointestinal disorders, especially focusing on pediatric functional abdominal pain disorders, functional constipation and infantile colic.

## 2. Intestinal Microbial Colonization in Early Life

Neonatal gut colonization is a stepwise process which is affected by genetic and maternal influences and, perhaps more profoundly, by environmental and dietary exposures. Recent reports from clinical and experimental studies suggest that intestinal colonization may begin already during fetal life by microbes present in the intrauterine environment [1,2]. These findings, while extremely interesting, need further confirmation in large clinical studies with methodological rigor to exclude the possibility of contamination during sample acquisition and all steps during sample preparation, processing and analysis.

Human neonates receive an important inoculum of colonizing microbes during vaginal delivery. While maternal vaginal microbes, primarily lactobacilli, transiently colonize the neonatal gut [3], it is evident that the maternal gut is the most important source of early colonizing bacteria to the neonate [4]. The significance of vaginal delivery to healthy gut colonization is underscored by data suggesting aberrant gut colonization patterns in infants born by caesarean section delivery as compared to those born vaginally [5]. After birth, gut colonization progresses in a stepwise manner and bifidobacteria soon dominate the gut microbiota of breastfed infants [6,7]. This is thought to primarily result from breast milk components, including glycoproteins and particularly human milk oligosaccharides (HMOs), which selectively enhance the growth of bifidobacteria [8]. This notion is supported by the fact that *Bifidobacterium longum* subspecies *infantis*, a microbe capable of utilizing a variety of HMOs, is practically universally encountered in breastfed infant microbiota throughout the world [9,10,11]. Moreover, the gut microbiota of infants fed with cow’s milk-based formula devoid of HMOs exhibits more diversity and a lower abundance of bifidobacteria [12]. While breastfeeding is associated with reduced risk of chronic non-communicable diseases including type II diabetes mellitus and obesity [13], the contribution of gut microbiota modulation to these health impacts is currently not known.

The infant and child gut microbiota gradually shifts to resemble that of adults and a stable, adult-like gut microbiota is thought to be established by the age of 2–3 years. The introduction of solid foods and particularly the cessation of breastfeeding are major driving forces of gut microbiota maturation [4]. It is noteworthy, however, that the mature gut microbiota exhibits considerable differences depending on geographical area [14]. The contribution of dietary practices most likely outweighs the effect of genetic differences in explaining this phenomenon. Throughout the maturation process, detrimental exposures and particularly antibiotic use may cause profound disturbances in gut microecology. The potential clinical significance of these temporary perturbations is illustrated by epidemiological studies suggesting an association between early-life antibiotic exposure and chronic disorders including overweight and obesity, asthma and inflammatory bowel disease [15].

## 3. Functional Gastrointestinal Disorders and Gut Microbiota

Since the early 1990s, the Rome foundation, a group of experts in functional gastrointestinal disorders (FGIDs), has collected the summary of knowledge among the FGIDs into the Rome criteria [16]. The most recent version, the Rome IV criteria, categorizes FGIDs among children and adolescents into three main classes based on the prime symptoms, i.e., functional nausea and vomiting disorders, functional abdominal pain disorders and functional defecation disorders. Functional nausea and vomiting disorders include cyclic vomiting syndrome, functional nausea and vomiting, rumination syndrome, and aerophagia. Functional abdominal pain disorders are classified into four groups: functional dyspepsia, irritable bowel syndrome (IBS), abdominal migraine, and functional abdominal pain (FAP)—not otherwise specified, whereas functional constipation and non-retentive fecal incontinence belong to the functional defecation syndromes [17].

According to the Rome IV criteria, infant colic is recurrent and presents with prolonged periods of crying, fussing or irritability in otherwise healthy infants under the age of 5 months. Colic crying resolves by the first five months of life and occurs without obvious cause and cannot be prevented or resolved [18]. However, the most widely accepted definition was penned by Wessel in 1954 as “paroxysms of irritability, fussing or crying lasting for a total of more than three hours a day and occurring on more than three days in any one week” in an otherwise healthy and thriving infant (19).

Since the Rome criteria are based on a systemic review of the literature and are widely adopted, we decided to concentrate here on only randomized clinical probiotic studies where the criteria have been used. However, in most of the colic studies, the “Wessel rule of three” has been used as the diagnostic criteria of infantile colic [19], and thus we included studies using those criteria as well. Moreover, the focus is on functional abdominal pain disorders, functional constipation and infantile colic since clinical trials in the field have been done almost exclusively with children with those disorders, as recently systemically reviewed [20,21]. In addition, in this review, we focused on the studies where these disorders were the primary outcome.

Given the association between early gut microbiota composition and chronic disease later in childhood, it is intriguing to hypothesize that disturbances in gut colonization might also play a role in the etiology and pathogenesis of childhood functional gastrointestinal disorders. The data regarding gut microbiota perturbations related to irritable bowel syndrome (IBS) and other functional gastrointestinal disorders in adults are not easy to interpret due to discrepant findings, despite attempts to adhere to generally accepted diagnostic criteria. The extrapolation of results obtained from an adult population to apply to children should always be done with caution. Only a few studies have systematically investigated gut microbiota composition or activity in children with functional gastrointestinal disorders.

In a case-control study of 22 school-aged children with IBS as defined by the Rome III criteria and 22 healthy controls, a fecal microbiota analysis by sequencing the 16S ribosomal RNA gene revealed that IBS was associated with a greater relative abundance of Proteobacteria, and particularly Gammaproteobacteria [22]. Rigsbee and colleagues [23] reported significant differences in gut microbiota composition between 22 school-aged children newly diagnosed with diarrhea-prominent IBS (IBS-D) fulfilling the Rome II criteria and 22 healthy children. Using several molecular methods (microarrays, 16S rRNA gene sequencing, quantitative PCR and fluorescent in situ-hybridization) they showed differing abundances of several bacterial genera between children with and without IBS-D. In contrast, no significant differences in fecal microbiota profiles or relative abundances of specific taxa were detected between 76 children aged between 4 and 17 years with functional constipation as defined by the Rome III criteria as compared to 61 healthy children of similar age [24]. Nonetheless, the children with functional constipation could be distinguished from the healthy matched controls by the ridge regression analysis of the fecal microbiota.

Whilst some studies suggest gut microbiota differences between pediatric patients with IBS and healthy children, it is not at all certain whether the gut microbiota plays a causal role in the pathogenesis of IBS. We are not aware of any reports with fecal samples obtained before the onset of IBS. However, a recent register-based study of more than 2 million individuals, of whom more than 14,000 had been diagnosed with IBS [25], suggests that caesarean section delivery is associated with a slightly increased risk of developing IBS (adjusted odds ratio (OR) 1.09, 95% confidence interval (CI) 1.03–1.16). This increase in risk, albeit small on an individual level, may at least in part be attributable to the aberrant gut colonization associated with caesarean section delivery. More circumstantial evidence for the connection between early gut microbiota perturbations and later functional gastrointestinal disorders may be drawn from a birth cohort study of more than 2700 children from Sweden [26], in which antibiotic use in the first or second year of life was associated with increased risk of recurrent abdominal pain in later childhood in girls. However, the association was not detected in boys.

The most comprehensive data on the association between gut microbiota alterations and functional gastrointestinal disorders are currently those regarding infantile colic. As early as 1994, Lehtonen and colleagues have reported based on culture methods that infants with colic were more often colonized by clostridia than healthy age-matched control infants, and that the difference was no longer detectable later at the age of three months [27]. Through the use of a modern microarray method in a case-control study of 12 infants with colic and 12 healthy age-matched controls with serial fecal samples, de Weerth and co-workers demonstrated that gut microbiota alterations are detectable already in the first weeks of life in infants who later develop colic [28]. Colic was specifically associated with the enrichment of Proteobacteria including *Escherichia*, *Klebsiella* and *Pseudomonas*, whereas the phyla Firmicutes and Actinobacteria were more prevalent in infants who did not develop colic. A decreased abundance of Actinobacteria and especially Bifidobacteria in the feces of infants with colic was recently confirmed by the 16S rRNA gene sequencing of fecal samples obtained from 37 infants with colic and 28 healthy controls [29]. Taken together, these data suggest not only that infantile colic is associated with altered gut microbiota composition but also that aberrant gut colonization precedes and may be causally related to the development of colic. This notion is corroborated by a recent report suggesting that maternal intrapartum antibiotic treatment, which is known to affect neonatal gut colonization, is more prevalent in infants who later develop colic [30].

## 4. Probiotics

The potential association between gut microbiota perturbations and functional gastrointestinal disturbances in children opens interesting therapeutic and preventive possibilities. The modification of the gut microbiota composition and activity by dietary interventions is currently an active area of research. Probiotics are one of the most commonly used treatment modalities.

Probiotics have been defined as live micro-organisms that, when administered in adequate amounts, confer a health benefit to the host [31]. It is important to note that, in order to be named a probiotic, the microbe in question must have evidence-based health effects. It is equally important to realize that probiotic effects are strain and species-specific. Clinical or mechanistic probiotic effects cannot be extrapolated to apply to other, even closely related microbes. This should also be borne in mind when devising or interpreting systematic reviews and meta-analyses of clinical probiotic studies.

The mechanisms of action of probiotics appear to be complex. It is often assumed that probiotics function by modulating the gut microbiota, but the evidence for this conjecture is sparse and the definition of probiotics cited above makes no reference to the gut microbiota. Specific probiotics have been shown to effective in reducing the risk and treatment of gastrointestinal disorders such as childhood infectious diarrhea [32,33], but it is not evident that these beneficial effects entail an impact on gut microecology. Moreover, there are clinical trials indicating that probiotic intervention on the pregnant and breastfeeding mother significantly reduces the occurrence of atopic dermatitis in high-risk infants with no effect on the infant gut microbiota composition [34,35]. Intriguingly, clinical and experimental studies have demonstrated that specific probiotics have direct effects on host physiological processes involving digestion and gut barrier function, immune responses, metabolism, nociception and behavior. It is therefore not surprising that probiotics have in some clinical trials shown clinical benefit in reducing the risk of diseases such as respiratory tract infections or otitis media [36], which have little to do with the gut microbiota. Based on all of this, it is paramount that determining the efficacy of probiotic interventions should be based on clinical criteria and not surrogate outcomes such as effects on gut microbiota.

### 4.1. Probiotics in the Management Pediatric Functional Abdominal Pain Disorders

Several clinical observations suggest that dysbiosis is a hallmark of IBS. First, symptoms in a substantial proportion of IBS patients are preceded by gastroenteritis or a round of antibiotics [37,38]. Moreover, rifaximin, the nonabsorbable antibiotic, has been shown to be effective in the treatment of adult patients with diarrhea-predominant irritable bowel syndrome (IBS-D) [39], although it was ineffective in children with chronic abdominal pain [40]. Indeed, gut microbiota alterations have found both in adults (reviewed in [41]) and children (reviewed above), thus offering a rationale for the therapeutic manipulation of gut microbiota in this group of patients.

*Lactobacillus reuteri* DSM 17938 has been the most widely studied probiotic in the field. Its effect has been investigated in 5 randomized clinical studies in children with FAP or IBS. In the first study, Romana et al. [42] compared the *Lactobacillus reuteri* with a placebo in 60 children. A significant reduction in pain intensity was found only in the probiotic group whereas a comparable significant reduction in pain frequency was shown in both groups. However, all these data were only graphically shown, without numeric presentation, limiting the interpretation of the findings [43]. Eftekhari et al. [43] did not find any significant differences between probiotic and placebo groups in severity of pain in 80 children with FAP despite a similar significant decrease within the groups as compared to the baseline. These studies both consisted of four weeks of intervention and follow-up.

Weizman et al. [44] evaluated the effect of *Lactobacillus reuteri* DSM 17938 with placebo in 101 children with FAP. At the end of the 4-week intervention, both the frequency and severity of pain were significantly lower in the probiotic group than in the placebo arm. After the 4-week follow-up, only the latter difference remained significant between the groups [44]. Jadresin et al. [45] studied *Lactobacillus reuteri* DSM 17938 in comparison to placebo in 55 children with FAP or IBS during a 16-week trial. Children in the probiotic group had more days without pain as compared to the placebo group during the study period. The intensity of pain was also less severe in the second and fourth month among the former group. However, absence from school or activities did not differ between the groups [45]. In the most recent study, Maragkoudaki et al. [46] compared *Lactobacillus reuteri* DSM 17938 to placebo in 54 children with FAP. Both the probiotic and placebo significantly reduced pain intensity and frequency from the baseline, but there was no significant difference between the groups. In addition, absence from school and use of analgesics were comparable between the groups [46].

The use of *Lactobacillus rhamnosus* GG in the management of pediatric FGIDs has been evaluated in three randomized clinical studies [17]. Bauserman et al. [47] found no difference in the change of abdominal pain severity between probiotic and placebo groups in 64 children with IBS. The number of responders was also similar between the groups. Abdominal distension was the only remaining symptom which was significantly less often present in the probiotic than the placebo group at the end of the 6-week study [47]. Gawronska et al. [48] investigated the effect of *Lactobacillus* GG versus placebo in 20 children with functional dyspepsia, 37 children with IBS and 47 children with FAP. Comparable amounts of patients (25% in probiotic and 9% in placebo group) reported no pain at the end of the 4-week study period. However, IBS patients receiving the probiotic were significantly more often without pain than patients on placebo (33% versus 5%) [48]. In the largest trial so far, Francavilla et al. [49] compared *Lactobacillus* GG versus placebo in 83 patients with IBS and 58 patients with FAP. They found that after a 4-week run-in and 8-week intervention, both pain intensity and frequency were significantly smaller in children with probiotic than those with placebo. These differences remained stable during the 8-week follow-up. Moreover, treatment was more often successful (i.e., at least 50% decrease both in pain intensity and frequency from the baseline) in the probiotic than the placebo group (72% vs. 53%) [49].

In addition to the above-mentioned studies, three trials have been conducted where other probiotic strains or combination of strains have been tested against placebo in children with functional abdominal disorders. Guandalini et al. [50] evaluated VSL#3 (a mixture of 8 strains) versus placebo in a crossover study of 67 children with IBS. Abdominal pain had decreased in both groups by the end of the 6-week intervention, but significantly more in the VSL#3 group. At week 6, the last week of the intervention, disruption of family life was assessed to be decreased more in the probiotic than placebo group [50]. Basturk et al. [51] investigated the effect of *Bifidobacterium lactis* B94 versus prebiotic inulin versus a synbiotic (inulin and *Bifidobacterium lactis* B94) in 71 children with IBS. The resolution of all symptoms during a 4-week trial was found in comparable amounts in the probiotic (39%) and synbiotic (29%) groups, but less often in those on inulin (12%). Giannetti et al. [52] studied a mixture of *Bifidobacterium infantis* M63, *Bifidobacterium breve* M16-V and *Bifidobacterium longum* BB36 in a crossover study of 50 children with IBS and 28 children with functional dyspepsia. They reported that abdominal pain disappeared significantly more often in the probiotic than placebo group in patients with IBS but not in those with functional dyspepsia. Again, quality of life improved significantly more often only in IBS patients on probiotics as compared to the same patients on placebo [52].

### 4.2. Probiotics in the Management of Pediatric Functional Constipation

Banaszkiewicz and Szajewska [53] investigated the effect of *Lactobacillus rhamnosus* GG versus placebo as an adjunct to lactulose in 84 children with constipation. Treatment success (at least 3 spontaneous bowel movements per week) was comparable between the groups both at the end of the 12-week intervention and 12 weeks later. Bu et al. [54] compared *Lactobacillus casei rhamnosus* Lcr35, magnesium oxide and placebo in 45 children with constipation in a 4-week trial. Lactulose and glycerin enema were allowed if stool passage was not noted for 3 and 5 days, respectively. The patients on probiotic and magnesium oxide had a higher defecation frequency, higher treatment success and fewer hard stools and less need for glycerin enema as compared to the placebo group [54].

Coccorullo et al. [55] studied the effect of an oil suspension with *Lactobacillus reuteri* DSM 17,938 or placebo in 44 infants with constipation in an 8-week intervention. Significantly more patients passed at least 5 stools a week in the probiotic than the placebo group at weeks 2, 4 and 8. Stool consistency did not differ between the groups [55]. Guerra et al. [56] evaluated a goat yogurt containing *Bifidobacterium longum* versus the yogurt alone in 59 children with constipation in a 10-week crossover intervention. When all the crossover data were analyzed, significant differences were observed between the groups in defecation frequency, defecation pain and abdominal pain. However, the authors did not state whether the difference was in favor of the probiotic goat yogurt or goat yogurt alone. In addition, all the results were presented graphically only [56]. Tabbers et al. [57] compared the effect of a fermented milk containing *Bifidobacterium lactis* DN-173 010 with a non-fermented milk-based dairy product in 159 constipated children. The rate of responders was similar in both groups. Moreover, stool frequency, pain during defecation, abdominal pain and bisacodyl use were comparable between the groups. However, flatulence was reported significantly less often during the 3-week study among those on the probiotic product [57].

Sadeghzadeh et al. [58] investigated the effect of lactulose plus a mixture of 7 probiotic strains versus lactulose plus placebo in 56 children with functional constipation during a 4-week intervention. Stool frequency and stool consistency improved in both groups, but significantly more so in those on lactulose plus probiotic. Russo et al. [59] studied polyethylene glycol 4000 plus a combination of three *Bifidobacteria* versus polyethylene glycol 4000 alone in 55 constipated children during an 8-week intervention. They reported that stool frequency and stool consistency improved in both groups as compared to baseline. However, no significant differences were detected between the groups [59]. Wojtyniak et al. [60] compared *Lactobacillus casei rhamnosus* LCR35 to a placebo in 94 children with constipation. Treatment success (at least 3 spontaneous stools per week without fecal soiling) was comparable between the groups although stool frequency was significantly lower in the probiotic group [60]. In the latest trial, the effect of *Lactobacillus reuteri* DSM 17938 and macrogol versus macrogol and matching placebo were studied in 129 constipated children for 8 weeks [61]. Stool frequency increased in almost all the patients and in comparable amount in both groups. Moreover, there were no significant differences between the groups in the number of patients with hard stools, painful defecation, large stools, fecal soling or abdominal pain [61].

In their comprehensive systemic reviews, Wegh et al. [20] and Wojtyniak and Szajewska [21] assessed the methodological quality and potential risks of bias of most of the studies reviewed above [42,43,44,45,46,47,48,49,50,51,52,53,54,55,56,57,58,59,60]. All in all, a relatively high risk of bias was found [20,21]. In addition, interventions and follow-up were short-term, the study populations were fairly small and heterogeneous as to their study design, probiotic strain and dose, duration of intervention and follow-up, and outcome measures. Therefore, no single strain or combination of strains can be recommended in the management of IBS, FAP or functional constipation in children. This is in accordance with a recent systematic review where potential dietary, pharmacological and psychological interventions of functional abdominal pain disorders in children were evaluated [62]. Probiotics were found to be effective if all the studies with different strains or combinations of them were pooled together: the odds ratio for improvement in pain was 1.61 (95% CI 1.15–2.27) for probiotics compared to placebo. When different strains were analyzed separately, the effect was not as clear, making a recommendation for clinical practice unjustified [62].

### 4.3. Probiotics in the Management of Infantile Colic

As altered gut microbiota, dysbiosis has been proposed to play a part in the pathophysiology of colic, probiotic bacteria have been suggested as a promising treatment for colic crying. Most intervention studies have examined the role of one specific probiotic, *Lactobacillus reuteri* DSM 17938. However, there are a handful of studies examining the role of other *Lactobacillus* spp. or mixture of different probiotics or synbiotics.

A recent systematic review and meta-analysis included altogether seven randomized controlled trials (471 participants) with a low risk of bias [63]. Five included randomized controlled trials (RCTs) involving 349 infants evaluated the effect of *L. reuteri* DSM 17938 at daily dose of 10^8^ colony-forming unit (CFU) given for 21 or 28 days [64,65,66,67,68]. *L. reuteri* was associated with treatment success (relative risk (RR) 1.67, 95% CI 1.10–2.81, number needed to treat (NNT) 5, 95% CI 4–8) and reduced crying times at the end of intervention (mean difference (MD)-49 min, 95% CI−66–33), nevertheless the effect was mainly seen in exclusively breastfed infants.

In accordance, an individual participant data meta-analysis (IPDMA), pooling raw data from four individual trials involving 345 infants [64,65,66,67] to create sufficient power for sub-group analysis, suggests that *L. reuteri* DSM 17,938 is effective in treating breastfed infants with colic, but not formula-fed infants [69]. The probiotic group was almost twice as likely than the placebo group to experience treatment success and averaged less crying and/or fussing time than the placebo group. Moreover, the intervention effects were dramatic in breastfed infants (NNT 2.6, 95% CI 2.0–3.6), but were insignificant in formula-fed infants. All the infants included in the meta-analysis were exclusively or predominantly breastfed, except the infants participating in the largest Australian trial, which included both breast and formula-fed infants. The gut microbiota composition of breastfed and formula-fed infants is distinct, and this might therefore explain the better effectiveness of probiotic intervention in breastfed infants. On the other hand, the superior effectiveness of *L. reuteri* in breastfed infants might also be explained by the direct effects of microbes or oligosaccharides in breast milk.

After publishing these two meta-analyses, two more RCTs with *L. reuteri* DSM 17938 in treating infantile colic in breastfed infants have been published [70,71]. A study with 60 colic infants showed *L. reuteri* significantly decreasing daily crying time during a 30-day intervention period [70], while a small trial with only 20 colic infants found no significant difference in daily crying time between the probiotic and placebo group [71].

Only one small RCT has examined the role of *Lactobacillus rhamnosus* GG (LGG) in treating infant colic during a 28-day intervention [72]. A study with 30 breast and formula-fed colic infants found no difference in daily crying time between infants receiving probiotic or placebo. However, it is interesting to note that the study suggested LGG to be effective by parental report, but not by the validated prospectively recorded Baby Day Diary. This finding emphasizes the importance of using uniform validated methods in measuring infant crying.

A recent RCT with a mixture of 8 different probiotic bacterial strains in 53 exclusively breastfed colic infants showed that the probiotic-mixture group had less crying per day than the placebo group at the end of 3-week treatment period [73]. In addition, a higher rate of infants from the probiotic-mixture group responded to treatment at end of the study. Interestingly, the probiotic intervention did not modify the gut microbiota composition compared to placebo in this study. However, the observation from a metabolomics perspective showed that the fecal molecular profile differed in connection with the treatment. Dupont et al. investigated the effect of a probiotic-supplemented (*L. rhamnosus* and *B. infantis*) and alpha-enriched formula versus standard formula on daily crying in 66 colic infants during a 1-month intervention period [74]. The study found no differences for crying duration between the probiotic and placebo groups.

There are two RCT investigating the role of synbiotics—the combination of probiotics and prebiotics—in treating infant colic. A trial with 50 breastfed colic infants receiving a synbiotic (containing *L. casei, L. rhamnosus, S. thermophiles, B. breve, L. acidophilus, B. infants, L. bulgariucs* and fructo-oligosaccharides) or placebo for 30 days demonstrated that treatment success was significantly higher in the synbiotic group compared with placebo at day 7 and 30 [75]. Another trial with 60 colic infants investigated the effect of intervention formula (containing *B. lactis* BB12, galacto-oligosaccaharides combined with reduced lactose and partial whey hydrolysate) on daily crying amount compared to standard formula [76]. During the 1-month intervention period, daily crying duration decreased significantly more in infants receiving synbiotics than standard formula.

Taken together, the role of mixtures of probiotics and synbiotics in the treatment of colic crying is promising, but still indefinite, due to the variation in the used probiotic strains, and more data is therefore needed before any conclusions can be drawn.

Thus far, two studies have examined the role of pro- and prebiotics in preventing infant colic as the primary outcome. A large RCT with 589 term infants studied the effect of *L. reuteri* 17938 or placebo during the first 90 days of life in preventing the onset of colic, gastroesophageal reflux and constipation [77]. The study concluded that daily administration of *L. reuteri* significantly reduces daily crying duration at an age of 1 monthcompared to the placebo, and the effect was sustained at 3 months. In addition, the number of regurgitations per day was significantly lower and the number of evacuations per day higher in infants receiving *L. reuteri* compared to placebo. Another randomized controlled trial of 94 preterm infants (gestational age 32–36 weeks) investigated the effects of *L. rhamnosus* GG versus galacto-ologosaccharides versus placebo in preventing infant colic during the first 2 months of life [78]. A total of 27 out of 94 infants were classified as excessive criers at the age of 2 months, while this was significantly less in the probiotic and prebiotic group than in the placebo group (19% vs. 19% vs. 47%).

## 5. Conclusions

Infantile colic seems to be both associated with and preceded by altered gut microbiota composition, suggesting that dysbiosis may be causally related to the development of the condition. As regards older children’s FGIDs, gut microbiota alterations have only been described in children with IBS, although their exact role in the pathogenesis remain unclear. So far, in addition to *Lactobacillus reuteri* DSM 17938 in the treatment of breastfed infant’s colic, no other probiotic or combination of them for the management of pediatric FGIDs can be recommended (Table 1). Further clinical studies among children with these disorders should preferably focus both on relevant clinical outcomes and gut microbiota composition and function together in order to get a more comprehensive view of the role of the gut microbiota in these common maladies.

## Figures and Tables

**Table 1:** Probiotics in children with functional gastrointestinal disorders. Summary of the RCT trials included in the review, quality of evidence and recommendation for clinical practice.

Disorder	Number of RCTs (No. of Children Altogether)					Quality of Evidence	Recommendation for Clinical Practice
	Probiotic Strain						
	*L. reuteri DSM17938*	*L. rhamnosus* GG	Other Single Strains	Mixtures of Probiotics	Synbiotics		
Treatment of functional abdominal pain disorders	5 RCT [42,43,44,45,46] (*n* = 350)	3 RCT [47,48,49] (*n* = 309)	1 RCT * [51] (*n* = 71)	2 RCT [50,52] (*n* = 145)	1 RCT * [51] (*n* = 71)	Low Not enough data High risk of bias in many of the studies [20,21]	No single strain or combination of strains can be recommended for the management of functional abdominal pain disorders [20,21]
Treatment of functional constipation	2 RCT [55,61] (*n* = 173)	1 RCT [53] (*n* = 84)	3 RCT [54,56,57,60] (*n* = 35)	2 RCT [58,59] (*n* = 111)		Low Not enough data High risk of bias in many of the studies [20,21]	No single strain or combination of strains can be recommended for the management of functional constipation [20,21]
Treatment of Infantile colic	7 RCT [64,65,66,67,68,70,71] (*n* = 429)	1 RCT [72] (*n* = 30)		2 RCT [73,74] (*n* = 119)	2 RCT [75,76] (*n* = 110)	Good Low risk of bias in most of the studies with *L. reuteri.* [63]	*L. reuteri* DSM 17938 at daily dose of 10^8^ CFU may be considered for the management of breastfed colic infants. Data on other probiotics or formula-fed infants are limited [63,69]
Prevention of Infantile colic	1 RCT [77] (*n* = 589)	1 RCT [78] (*n* = 94)				Moderate Low risk of bias Data mostly from one study	No single strain can be recommended for the prevention of infantile colic, although there are promising data on *L. reuteri* DSM 17938 [77,78]

RCT=randomized controlled trial, *L. Lactobacillus,* * Same RCT.
